# Perspective: Mexico’s Experience in Building a Toolkit for Obesity and Noncommunicable Diseases Prevention

**DOI:** 10.1016/j.advnut.2024.100180

**Published:** 2024-01-19

**Authors:** Juan A Rivera, Mónica Arantxa Colchero, Carolina Pérez-Ferrer, Simón Barquera

**Affiliations:** 1 Center for Population Health Research, National Institute of Public Health, Cuernavaca, Morelos, Mexico; 2 Center for Evaluation and Surveys Research, National Institute of Public Health, Cuernavaca, Morelos, Mexico; 3 Center for Nutrition and Health Research, National Institute of Public Health, Cuernavaca, Morelos, Mexico

**Keywords:** Mexico, nutrition policy, overweight, obesity, health taxes, front-of-pack warning labels, marketing regulations, school food policies, dietary guidelines

## Abstract

Noncommunicable diseases (NCDs) are a leading cause of death and disability worldwide, with a higher risk of death in low- and middle-income countries. Diet and excess weight are risk factors for NCDs. In Mexico, the prevalence of overweight and obesity increased dramatically in the last 30 y and is among the highest in the world. To address this public health problem, governments and public health professionals have several policy instruments available. In this study, we present the policy instruments currently approved in Mexico, which include fiscal, informational, and authoritative tools that aim to improve the food environment and promote healthy behaviors (taxes, school food guidelines, front-of-pack labeling, marketing regulations, and dietary guidelines). These types of interventions are important in regions like Latin America, where social inequities and poor access to information are common, and individual healthy choices are often limited. These interventions target the environments in which individuals live, study, work, and seek entertainment, while limiting access to unhealthy choices and offering information to promote healthy alternatives. The Mexican experience in design, implementation, and evaluation of policies to improve the food environment can be useful for other low- and middle-income countries facing similar challenges.

**Statement of significance**

There are a number of cost-effective policy tools that help improve the food environment and prevent noncommunicable diseases (NCDs). In countries with vast inequalities and with food systems incentivized to produce unhealthy options, a mix of policy tools is needed to effectively modify food environments, promote healthier food choices, and prevent and control the increasing trends in obesity and NCDs. In addition, more complex, global initiatives are required to achieve the transformation of the food system.

## Introduction

Noncommunicable diseases (NCDs) are leading causes of death and disability worldwide, with a higher risk of death in low- and middle-income countries. Poor diets and obesity are among the most important risk factors for NCD’s and premature deaths and they are modifiable.

The prevalence of obesity in Mexico increased dramatically in the last 30 y and is among the highest in the world. Seventy-five percent of adults and ∼40 % of children and adolescents (5–19 y of age) are overweight or obese [1,2]. NCD’s are also common, for example, the prevalence of diabetes and hypertension in Mexican adults were 15.7 % and 30.2 % in 2020, respectively [3,4]. A large part of this widespread weight gain and high NCD prevalence is attributed to poor diets, specifically increases in the consumption of ultra-processed foods, such as salty and sugary snacks, sugar-sweetened beverages (SSB), and other caloric beverages [5] which displace healthy foods [6]. These unhealthy products account for ∼30 % of total energy intake [6]. SSBs alone provide on average 9.8 % of the total energy intake and are the main source (69 %) of added sugar [7]. In contrast, the intake of healthy foods (vegetables, fruits, legumes, nuts, and whole grains) is much lower than recommended [7]. To address this public health problem, governments and public health professionals have resorted to diverse policy instruments that range from regulatory and binding to voluntary guidelines.

## Policy Instruments for the Prevention of Obesity and Diet-Related NCDs

Decision makers, civil society and academia interested in the prevention of obesity and diet-related NCDs have a range of cost-effective policy instruments available for this purpose [8,9]. From a public policy perspective, policy instruments are generic tools of government action used to achieve a certain policy goal [10]. These instruments can be classified according to the level at which they intervene and to their obligatory compared with voluntary nature [10].

Information-based tools involve communicating information to target groups, which can be consumers or producers, with the expectation that these will alter their behavior [10]. For example, using mass-media campaigns to encourage healthy eating, front-of-pack warning labels to discourage ultra-processed food purchases, or one-to-one nutrition counseling to lose weight. These policy tools require an important amount of individual effort, and their health impact is limited in contexts where the environment is very unhealthy or where there are wide social inequalities that act as barriers for the adoption of healthy choices [11].

Financial implementation tools include financial incentives to encourage certain behaviors aligned with policy goals or the imposition of financial costs to discourage unaligned behaviors [10]. Some examples are conditional cash transfer programs, excise taxes, subsidies and vouchers, to name a few, that have been used to achieve public health goals.

Authoritative implementation tools involve the design and approval of binding rules which aim to steer behaviors toward policy goals. These are obligatory policy instruments and can take the form of standards, permits, norms, laws, or executive orders. These instruments can be enforced by the state, using real or perceived sanctions [10].

Finally, organizational implementation tools, which have not been used so far in the Mexican experience, are governing instruments that rely upon the use of government institutions and personnel to affect the production, distribution, and consumption of goods and services [10].

From a public health perspective, we tend to be interested in making the “healthy choice the default choice.” That is, modifying the environments in which individuals live, study, work, and seek entertainment to make them more conducive to healthy choices. This can be achieved using a mix of the policy instruments described above. Ultimately, policymakers must select tools according to what is considered feasible or possible in a given context.

In this perspective piece, we discuss the policy instruments used in Mexico to help prevent and control obesity and other diet-related NCDs. Given the multifactorial nature of obesity and NCDs, to revert the current epidemics, the implementation of a comprehensive set of policies is required, rather than isolated interventions. These policies should emphasize the transformation of food systems (including production, processing, distribution, and the food environments) to achieve healthy food patterns in the population. In Mexico, policies so far have focused mainly on modifying the food environment. Successful experiences with policy instruments targeting the food environment can become part of an NCD and obesity prevention policy toolkit in any country facing similar challenges.

**FIGURE 1:**
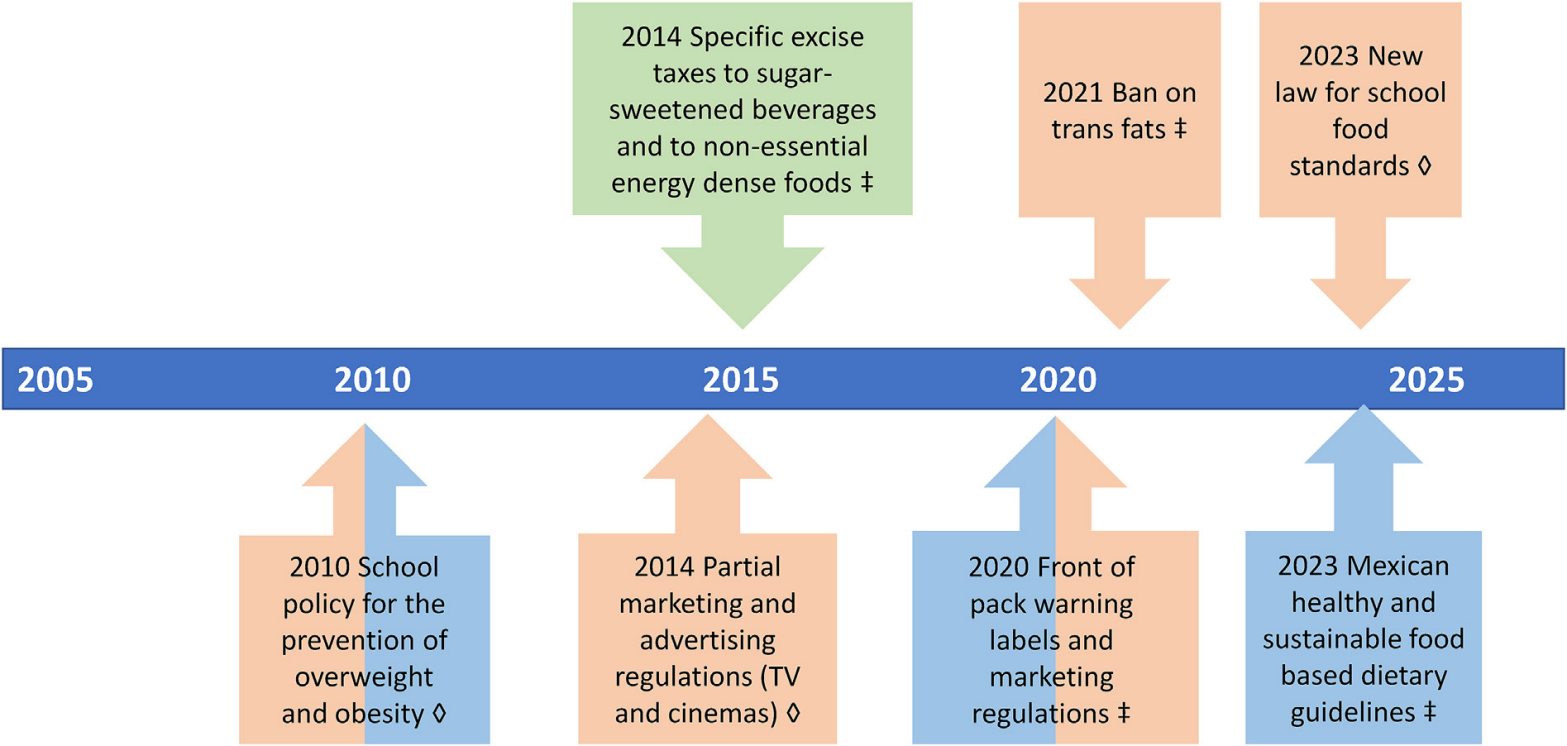
Timeline of policy instruments implemented in Mexico for the prevention of obesity and diet-related NCDs. Green: financial policy tool; orange: authoritative policy tool; and blue: informational policy tool. ◊ Policy instruments targeted at children and adolescents. ‡Population level policy instruments. NCD, noncommunicable disease.

Figure 1 illustrates the timeline and variety of policy instruments implemented in Mexico. They include school food standards approved in 2010; a specific tax for SSBs and an ad valorem tax on nonessential energy-dense foods (2014); partial marketing and advertising regulations (2014); front-of-pack warning labels (2020); a ban on trans fats (2021); new healthy and sustainable dietary guidelines for the Mexican population (2023); and more binding food standards for schools (2023). They target 5 of the 7 dimensions of the food environment identified in the International Network for Food and Obesity/noncommunicable disease Research, Monitoring and action Support (INFORMAS) framework: food provision within schools, food prices, food marketing, food labeling, food composition, and dietary guidelines [12]. INFORMAS is a global network of organizations and researchers that aims to monitor, benchmark, and support public and private sector actions, providing evidence to advocate for healthier food environments [12]. The color of the boxes in Figure 1 classifies policies into the taxonomy described earlier. Each of these policies will be described in more detail in the following sections beginning with the financial policy tools (the most successful and better documented at this point), followed by the authoritative tools and informational instruments.

### Implementation and Evaluation of Taxes in Mexico

Mexico was one of the first countries that implemented country-wide Pigouvian taxes to SSBs and to nonessential energy-dense food in 2014. Pigouvian taxes are designed to reduce negative externalities such as healthcare costs associated with the consumption of goods that harm health [13]. However, the negative externality has been harder to justify for SSBs compared with tobacco and alcohol taxes, particularly as the industry promotes individual responsibility [14]. Today, economists and international organizations refer to taxes as a policy to correct for externalities [15,16]. Fiscal policies are promising as implementation costs are low because most countries have the infrastructure to collect other taxes [17].

In Mexico, specific taxes need approval from Congress. Given the high consumption of SSBs and powerful sectors opposing their implementation, the task was challenging. The process required a strategic partnership of key actors who generated and compiled evidence about the potential health benefits of a tax and disseminated this evidence to critical stakeholders and to the public. Thus, academia generated or summarized evidence that showed *1*) a high prevalence of diet-related NCDs and increasing trends in the prevalence of obesity and diabetes; *2*) the link between SSB intake and risk of obesity and diabetes; *3*) high SSB consumption and contribution to total energy intake in the Mexican diet; *4*) the high costs of obesity and diabetes; *5*) estimation of own and cross price elasticities of the demand of SSBs; and *6*) potential revenues from different tax levels. Likewise, advocacy organizations raised awareness among the public and fostered public demand for the tax and lobbying organizations who used the evidence from academia to inform members of Congress about the health, social, and economic benefits of taxing SSBs. The first proposal was justified by the previously mentioned evidence and supported by academia and civil society. It came from an opposition party senator who agreed to champion a 2 peso/L SSB tax (20 % of the price). However, the approved proposal (a $1 peso/L tax) was a compromise proposed by the executive branch, whose party had the majority in Congress. In addition, an excise ad valorem tax of 8 % on nonessential foods with energy density ≥275 kcal/100 g, which were also high in key nutrients associated with ill health (unhealthy foods), was also approved [18]. Both taxes were implemented in 2014. Because of space limitations, we emphasize the discussion on the SSB tax, given that more evidence was used for its justification and its evaluation has been more thorough.

The fiscal instrument approved was an excise tax of 1 peso/L on all nonalcoholic beverages with added sugar, excluding 100 % juices and beverages with artificial sweeteners. Earmarking the tax to obesity and NCD prevention strategies was discussed but the Secretary of Finance did not approve this budgeting practice. Instead, a provision was included to request the Secretary of Finance to use part of the revenues for obesity prevention. However, monitoring this provision has proved to be very difficult. The impact of a tax on consumption relies mainly on increases in prices. The magnitude of this reduction depends on several factors: price elasticities (how consumers respond to increases in prices), pass-through prices (how much of the tax is reflected in the price), and industry responses. The pass-through prices were heterogeneous. It was complete in urban areas (prices increased by the amount of the tax), and higher for noncarbonated beverages and smaller package sizes than for carbonated beverages and larger package sizes [19]. However, the pass-through prices were incomplete in rural areas (prices increased less than the amount of the tax) [20].

The evaluation of the policy included a study using a commercial dataset that estimated purchases of taxed and untaxed beverages before and after the tax implementation, adjusting for previous trends (2012–2013). Compared with predicted (counterfactual) purchases based on past trends, posttax purchases of taxed beverages showed an average reduction of 7.6 % over 2014–2015. An increase in purchases of water and other nontaxed beverages was also documented [21]. Similar results were found in a study using household purchases at the national level [22]. Higher reductions were found among households of low socioeconomic status [21], among those consuming higher amounts [23], in households with children and adolescents and among urban dwellers [22]. Larger reductions were found in a study relying on a before-after tax implementation [24].

Most of these studies show increases in the demand for untaxed beverages, mostly bottled water (the data lack information on potable water) [[22], [23], [24]], suggesting that the population substituted for a healthier beverage option.

A comparison of national dietary surveys before and after the implementation of the SSB and unhealthy food taxes showed a decrease in the contribution to the total energy of the taxed items and in the content of unhealthy nutrients, particularly added sugars, indicating improvements associated with taxes [25].

In addition to modeling studies showing the potential benefits of the SSB tax on obesity and NCDs [26], a study using secondary data found positive impacts on oral health associated with the fiscal policy implemented in 2014 [27]. Concerns about the negative impacts of the tax on employment were challenged by a study that found no significant reductions in a number of employees in the beverage industry or in commercial establishments [28].

Should the SSB tax increased to get more benefits? Based on a recent study that estimates how much taxes represent of final retail prices, the excise tax share for Mexico is low: 5.3 % compared with Chile: 15.1 % [29]. Given the low tax share and the potential benefits for consumption, taxes should increase at an optimal rate to maximize benefits (consumption and health) and minimize negative impacts (employment and income).

Evaluation of the unhealthy foods tax documented an average reduction of 5.3 % in purchases relative to what would have been expected based on pretax (2012–2013) trends, with no corresponding change in purchases of untaxed foods [18].

More than 60 countries/cities in the world have implemented SSB excise taxes after the Mexican experience with different designs and amounts. The large variation of designs from tiered taxes to sugar content, excise taxes to volume and ad valorem taxes provide a rich natural experiment to compare the effectiveness of different designs. A recent systematic review and meta-analysis of 62 studies examining the effects of SSB taxes worldwide shows that taxes were associated with higher prices and lower sales of taxed beverages; pass-through prices of 82 % (average percent of the tax reflected in the price) and a reduction in the demand for SSB of 15 % were documented [30].

### Front-of-Pack warning labels

Mexico has approved a regulation for packaged foods, implemented in 2020, that includes warning labels on ultra-processed packaged foods and beverages with excessive amounts of critical ingredients (sugar, saturated and trans fats, and sodium), as well as total energy according to the Pan-American Health Organization nutrient profile. Moreover, products with warning labels are not allowed to use cartoon characters and food claims. The regulation also made it mandatory to declare all types of sugar in descending order in the nutrient facts panel. In addition, it includes 2 warning legends regarding noncaloric sweeteners or caffeine, recommending that their consumption be avoided by children. This regulation was inspired by the Chilean system which was better understood by the Mexican population than the previous Guideline Daily Amount labels [31].

Although warning labels are a type of information-based tool aimed at modifying consumers’ behavior by altering the information environment, some aspects of this regulation are authoritative [10]. Labels are mandatory, not optional, and all packaged products must bear them. Furthermore, they are linked with marketing regulations which are binding and therefore must be observed by all food producers and food distributors in the country. Enforcement is strict and there are consequences for not complying; for example, companies may receive fines for violations.

Diverse evaluations in progress are documenting the impact of these policies in Mexico and elsewhere. An evaluation of the law in Chile mandating front-of-pack warning label (FPWL), restricting marketing, and banning school sales of unhealthy food has shown that in comparison with the counterfactual scenario, overall calories, sugar and sodium purchased declined by 3·5 %, 10·2 %, and 4·7 %, respectively, in the overall population and 23·8 %, 26.7 %, and 36·7 % among high-consumers [32]; another study showed reductions of 23.7 % in the consumption of SSBs [33]. Mexico is in the process of evaluating the FPWL. A recent modeling study estimated an average decline of caloric consumption of 36.8 kcal/d associated with the label implementation, which is projected to lead to a reduction of 1.3 million cases of obesity and savings of United States $1.8 billion in direct and indirect costs after 5 y [34].

Similarly to the case of SSB taxes, after showing positive preliminary results, the FPWL implemented first in Chile has been approved with variations in Uruguay, Peru, Mexico, Argentina, Colombia, and other countries [31]. This domino effect is in part due to strong international research and civil society organizations networks sharing information and collaboration such as COLANSA (https://colansa.org/).

### Regulation to the marketing and advertising of food products to children

Food and beverage marketing to children is widespread and its link to unhealthy dietary patterns is well described [35]. Efforts to regulate food advertising and marketing have been made in Mexico, but there is room for improvement. In 2009, a voluntary code designed and promoted by the food industry was implemented but had a very limited impact on the number of advertisements and nutrition quality of the foods being advertised [36,37]. Then, in 2014, a mandatory regulation came into place for television programming and cinemas. Unfortunately, the language used in the law was vague, did not establish a nutrition profile for food that could be advertised, regulations were restricted to certain time schedules, excluding times during evening and night when children are often watching TV; and the regulation was restricted to TV shows which are considered for children and excluded programs that are not supposed to be for them, but to which children are actually exposed, for example, sporting events and soap operas. Finally, adolescents were not considered in the mandatory regulations. Furthermore, because it was limited to television and cinemas, it left out digital marketing and other marketing strategies commonly used by the food industry, such as gifts and sponsorship of events [38]. Recent research from Mexico estimates that up to 70 % of children and adolescents are exposed to digital food marketing, with up to 47 food marketing exposures through their electronic devices per week. The great majority (>90 %) of foods being advertised through digital channels are considered unhealthy by the Mexican Nutrient Profile Model [39,40]. The public health community, including researchers and civil society organizations, have called for a more rigorous regulation that, at a minimum, includes adolescents as well as children, uses clear nutritional criteria for foods that can be advertised, is aligned with the Mexican FPWL Nutrient Profile [40] and has a broader reach in terms of marketing strategies [38].

Another area of concern is the marketing of commercial breastmilk substitutes and baby food. A growing body of research has documented how the International Code of Marketing of Breastmilk Substitutes is frequently violated in the country, with companies using powerful marketing strategies on the internet and social media and generating conflicts of interest with health professionals [41,42]. No mandatory regulation exists in the country. Therefore, it is essential to include actions promoted by the code in the national legal framework, to monitor compliance and to enforce sanctions for violations of the code.

### School food standards

In 2011, the Ministries of Education and Health, with support from academia, developed and approved evidence-based nutrition standards for foods and beverages sold in schools in the context of a wider policy to prevent childhood overweight and obesity [43]. Their aim was to decrease the availability of foods exceeding critical nutrients (fat, sugar, and salt) and energy content, while promoting the consumption of fruits, vegetables, and plain drinking water. The standards were unambiguous, with clear nutrition criteria for foods allowed and for the composition of meals such as school breakfasts and school lunches [43]. They also included guidelines for snacks brought from home and the creation of school committees with participation of parents, teachers, and authorities with the purpose of supervising the implementation of the regulations. The design of the policy was informed by the best available evidence and its approval, at the time, was a great win for the public health community.

These school standards were mainly intended to be an authoritative tool with a smaller component of an information-based instrument. However, they did not have coercive power (no sanctions for noncompliance) nor proper supervision (no budget for this purpose); therefore, they have not been well implemented [44,45]. Studies have documented high availability of foods that do not meet the standard’s nutrition criteria in schools and resistance to change foods brought from home that do not comply with guidelines [45]. Limitations of the policy included: lack of training and support for implementation actors, reliance on parents instead of school or local authorities to enforce compliance, low involvement of parents in supervision committees due to lack of time and other priorities, lack of concrete and measurable objectives for the policy, and absence of accountability mechanisms [46]. The implementation of the policy failed to convince parents about its importance and to keep them engaged [46]. As a result, school standards in Mexico have in fact served as an information-based tool for schools with very limited results.

The policy was revised in 2023 to make it more binding and received its final approval in November 2023 [47]. Rather than modifying the standards, the General Law for Education was revised. The key changes approved include regulation of publicity of unhealthy foods within schools and around them; linking the policy to the FPWL so that foods that can and cannot be sold are more easily identifiable, including ultra-processed foods sold in bulk; and making school Directors accountable for compliance. With these updates, the school standards, now part of the law, will overcome the main limitations for compliance that were previously mentioned.

### Mexican Healthy and Sustainable Food-Based Dietary Guidelines

**FIGURE 2:**
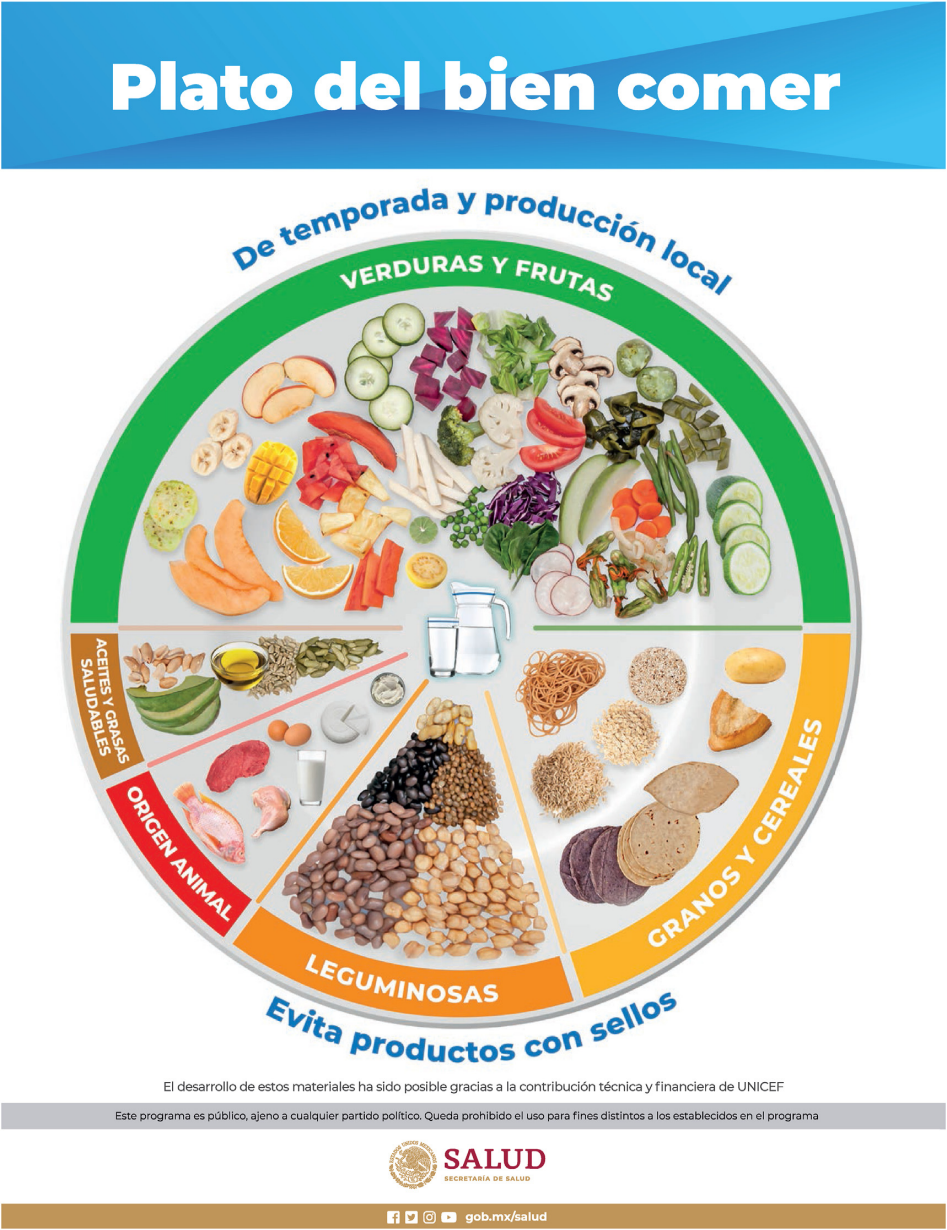
Mexico’s new eat-well plate.

**FIGURE 3:**
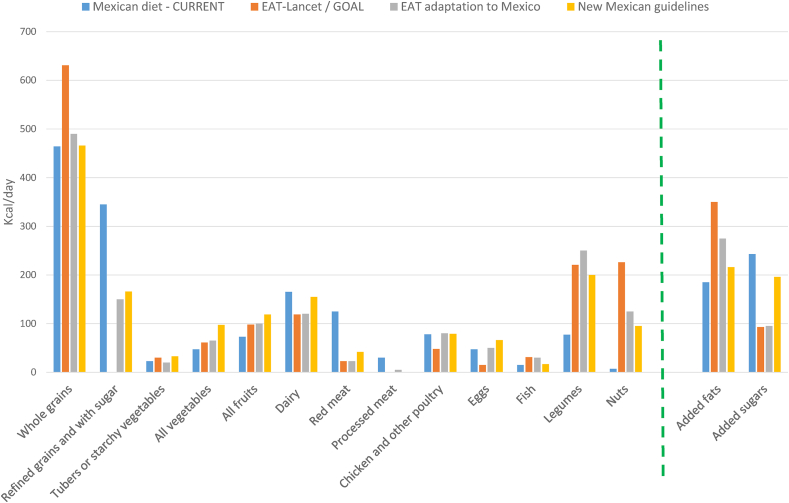
Comparison between the current Mexican diet, EAT-Lancet recommendations and the new healthy and sustainable food-based dietary guidelines. Prepared by the authors with information from El Poder del Consumidor [47], Mota-Castillo et al. [51], and Hernandez S. (unpublished).

The most recent effort to improve the population’s diet in Mexico was the development of new healthy and sustainable food-based dietary guidelines accompanied by a new “eat-well plate” (Figure 2). These guidelines adapt the EAT-Lancet food group targets in light of current dietary patterns in Mexico which are neither healthy nor sustainable [47,48]. Figure 3 compares the current Mexican diet to the recommended distribution of calories according to *1*) EAT-Lancet targets, *2*) an adaptation of the EAT-Lancet targets to Mexico, and *3*) the new healthy and sustainable dietary guidelines [[48], [49], [50]]. The new guidelines allow the consumption of certain foods that are not recommended by the EAT-Lancet targets (refined grains and cereals with added sugar) or allow intakes that are above (eggs which are popular and affordable) or below (nuts which are consumed in very modest amounts in Mexico) the EAT-Lancet targets. Thus, the guidelines represent interim dietary goals for the short and medium term, which are meant to be revised periodically as the current diet moves to the recommended intakes.

A healthy and sustainable diet has been shown to be 29 % more affordable than the current Mexican diet and 22 % more affordable than the current isocaloric Mexican diet [52]. The increased cost of fruits, vegetables, legumes, and nuts is offset by savings from a lower consumption of red meats, SSBs, and discretionary foods [52].

Guidelines such as these serve several purposes. They are an information-based tool to aid nutrition education but also, they are key for the development of public policy because they establish the recommended diet and therefore the goal for public health nutrition policies. The next steps for these guidelines include their dissemination to the population and identifying key policies and programs that should be revised to be aligned with the guidelines.

## Discussion

The process of achieving the policies described in this article required collaboration among academia, civil society, and the legislative or executive branches of the Government. The design, approval, and implementation processes of some of the policies, for example, the sugar-sweetened beverages taxes and the FPWL, were complex and took a long time, facing strong opposition from multinational ultra-processed food companies. The model of collaboration between academia and civil society resulted in the mobilization of public support and social pressure for policy action [53], which was key to success.

Mexico is in the process of building its policy toolkit (a mix of policy instruments) for obesity and NCD prevention. So far, policy instruments have evolved mostly (with a few exceptions) as independent tools in an incremental fashion because the opportunities provided by the policy environments in Mexico have favored the development of independent tools. However, most actors that have participated in the design and implementation of policies would have favored a customized package of interventions that created synergies. The assumption has been that each new policy in the toolkit improves the potential effects in an additive fashion. However, it is generally accepted that a combination of policy tools is expected to be more effective and efficient in achieving policy objectives than single instruments as long as the mix displays coherence, consistency, and congruence [10,54,55]. One advantage of having the components of the toolkit approved independently is the flexibility to improve and update them without undergoing a complex process such as the one needed to modify comprehensive national policies.

Recently, the opportunity has come to generate more synergies among policy tools. As mentioned earlier, the school food standards were recently revised and incorporated into the General Law for Education and explicit links with other regulations were approved, including mandating that foods with FPWL are banned from schools and cannot be marketed within schools and around them. Moreover, a bill initiative is under discussion in Congress to transform an intersectoral group for health, nutrition, environment, and competitiveness (GISAMAC, an acronym in Spanish) into a legal entity. This group aims to promote a fair, healthy, sustainable, and competitive food system. The group has been working informally (see below) but the lack of legal status has limited its effectiveness in the design and implementation of policies. The institutionalization of the intersectoral collaboration is an organizational implementation instrument*;* this policy tool has not been employed so far in the Mexican toolkit for obesity prevention.

Policy instruments implemented to date in Mexico have been mainly focused on changing the demand for food; however, they have had some effects on the supply side through the response of food industry to the regulations. For example, the FPWL and unhealthy food tax led to a reformulation of unhealthy products [56] to avoid the regulation and the school standards led to changing portion sizes of certain products that were allowed in smaller presentations.

Also, most of the Mexican policies specifically discourage the demand for unhealthy foods. The supply of healthy foods has received less attention from the public health nutrition community and is pending on the agenda. Although food supply policies exist, for example, corn subsidies, or guaranteed prices for certain crops, these are usually not aligned with nutrition goals [57]. Transformation of food systems to promote healthier diets, including agricultural, trade, and investment policies requires strong coordination, a shared vision among diverse sectors, and international governance, given the globalized economy [58]. As mentioned earlier, Mexico has taken the first step for this purpose creating GISAMAC; however, its reach has been limited and has yet to achieve tangible policies to align food production and sustainable nutrition goals. Some promising policy tools that could be explored include preferential procurement for school food (for example, the Brazilian experience [59]) or aligning production subsidies with population nutrition and sustainability goals. Mexico is leading an effort to develop a regional network for government cooperation to promote the food system transformation, with a focus on efforts to develop an ultra-processed food control framework and efforts to reduce the use of agrochemicals and promote agroecology.

Also pending on the agenda are systemic changes to address the commercial determinants of health. These include rebalancing power in favor of health and the common good, exposing conflicts of interest, and dealing with industry interference in policy making and implementation by promoting regulation of corporate practices that interfere with policy making. Industry interference has been documented in the design and implementation of SSB and unhealthy food taxes, school food regulations, and FPWL. Interference remains even after documenting the effectiveness of these policies, as in the case of SSB [60,61].

Other policies that are pending include regulations of marketing strategies directed to children in points of purchase [31] and digital spaces [39,62], the adoption and enforcement of the recommendations from the International Code of Marketing of Breast-milk substitutes from the World Health Organization, strengthening implementation of school food standards, and subsidies to healthier foods. Furthermore, a more explicit life-course perspective has been missing and some age groups have received less attention.

Obesity and NCDs are complex public health problems with extensive social and economic costs. To address them, a mix of policy instruments is needed to provide information and modify food systems, including the food environment, to align incentives toward healthier choices. We have presented Mexico’s successful experience with Pigouvian taxes, as well as incipient results for FPWL and other policies with varying degrees of success, and we have discussed lessons learned. We argue that these, together with other pending policies, have the potential to create healthier food environments and food systems. In turn, they can lead to healthier populations through the control and prevention of the increasing trend in obesity and diet-related NCDs in years to come.

## Author contributions

The authors’ responsibilities were as follows – JAR: conceptualization, writing original draft, review, and editing; MAC: writing original draft, review, and editing; CPF: writing original draft, review, and editing; SB: writing original draft, review, and editing; and all authors: read and approved the final version of this manuscript.

## Conflicts of interest

The authors report no conflicts of interest.

## Funding

The authors reported no funding received for this study.

## References

[1] Barquera S., Hernández-Barrera L., Trejo-Valdivia B., Shamah T., Campos-Nonato I., Rivera-Dommarco J. (2020). [Obesity in Mexico, prevalence andtrends in adults. Ensanut 2018–19]. Salud Publica Mex.

[2] Shamah-Levy T., Cuevas-Nasu L., Gaona-Pineda E.B., Gómez-Acosta L.M., Morales-Rúan M.D.C., Hernández-Ávila M. (2018). [Overweight and obesity in children and adolescents, 2016 Halfway National Health and Nutrition Survey update]. Salud Publica Mex.

[3] Basto-Abreu A.C., López-Olmedo N., Rojas-Martínez R., Aguilar-Salinas C.A., De la Cruz-Góngora V.V., Rivera-Dommarco J. (2021). Prevalence of diabetes and glycemic control in Mexico: national results from 2018 and 2020. Salud Publica Mex.

[4] Campos-Nonato I., Hernández-Barrera L., Oviedo-Solís C., Ramírez-Villalobos D., Hernández-Prado B., Barquera S. (2021). Epidemiología de la hipertensión arterial en adultos mexicanos: diagnóstico, control y tendencias. Ensanut. Salud Publica Mex.

[5] Hall K.D., Ayuketah A., Brychta R., Cai H., Cassimatis T., Chen K.Y. (2019). Ultra-processed diets cause excess calorie intake and weight gain: an inpatient randomized controlled trial of ad libitum food intake. Cell Metab.

[6] Marrón-Ponce J.A., Tolentino-Mayo L., Hernández F.M., Batis C. (2018). Trends in ultra-processed food purchases from 1984 to 2016 in Mexican households. Nutrients.

[7] Rivera J.A., Pedraza L.S., Aburto T.C., Batis C., Sánchez-Pimienta T.G., González de Cosío T. (2016). Overview of the dietary intakes of the Mexican population: results from the National Health and Nutrition Survey 2012. J. Nutr..

[8] Gortmaker S.L., Long M.W., Resch S.C., Ward Z.J., Cradock A.L., Barrett J.L. (2015). Cost effectiveness of childhood obesity interventions: evidence and methods for CHOICES. Am. J. Prev. Med..

[9] Goryakin Y., Aldea A., Guillemette Y., Lerouge A., Feigl A., Devaux M., OECD (2019). The heavy burden of obesity: the economics of prevention.

[10] Howlett M. (2019).

[11] Kite J., Grunseit A., Bohn-Goldbaum E., Bellew B., Carroll T., Bauman A. (2018). A systematic search and review of adult-targeted overweight and obesity prevention mass media campaigns and their evaluation: 2000–2017. J. Health Commun..

[12] Swinburn B., Sacks G., Vandevijvere S., Kumanyika S., Lobstein T., Neal B. (2013). INFORMAS (International Network for Food and Obesity/non-communicable diseases Research, Monitoring and Action Support): overview and key principles. Obes. Rev..

[13] Brownell K.D., Farley T., Willett W.C., Popkin B.M., Chaloupka F.J., Thompson J.W. (2009). The public health and economic benefits of taxing sugar-sweetened beverages. N. Engl. J. Med..

[14] Brownell K.D., Kersh R., Ludwig D.S., Post R.C., Puhl R.M., Schwartz M.B. (2010). Personal responsibility and obesity: a constructive approach to a controversial issue. Health Aff. (Millwood)..

[15] World Bank Group (2020).

[16] Allcott H., Lockwood B.B., Taubinsky D. (2019). Should we tax sugar-sweetened beverages? An overview of theory and evidence. J. Econ. Perspect..

[17] Popkin B.M., Ng S.W. (2021). Sugar-sweetened beverage taxes: lessons to date and the future of taxation. PLOS Med.

[18] Batis C., Rivera J.A., Popkin B.M., Taillie L.S. (2016). First-year evaluation of Mexico’s tax on nonessential energy-dense foods: an observational study. PLOS Med.

[19] Colchero M.A., Salgado J.C., Unar-Munguía M., Molina M., Ng S., Rivera-Dommarco J.A. (2015). Changes in prices after an excise tax to sweetened sugar beverages was implemented in Mexico: evidence from urban areas. PLOS ONE.

[20] Colchero M.A., Zavala J.A., Batis C., Shamah T., Rivera J.A. (2017). Cambios en los precios de bebidas y alimentos con impuestos en áreas rurales y semirrurales de México. Salud Publica Mex.

[21] Colchero M.A., Rivera-Dommarco J., Popkin B.M., Ng S.W. (2017). Mexico, evidence of sustained consumer response two years after implementing a sugar-sweetened beverage tax. Health Aff. (Millwood)..

[22] Colchero M.A., Molina M., Guerrero-López C.M. (2017). After Mexico implemented a tax, purchases of sugar-sweetened beverages decreased and water increased: difference by place of residence, household composition, and income level. J. Nutr..

[23] Ng S.W., Rivera J.A., Popkin B.M., Colchero M.A. (2019). Did high sugar-sweetened beverage purchasers respond differently to the excise tax on sugar-sweetened beverages in Mexico?. Public Health Nutr.

[24] Pedraza L.S., Popkin B.M., Batis C., Adair L., Robinson W.R., Guilkey D.K. (2019). The caloric and sugar content of beverages purchased at different store-types changed after the sugary drinks taxation in Mexico. Int. J. Behav. Nutr. Phys. Act..

[25] Batis C, Castellanos-Gutiérrez A, Sánchez-Pimienta TG, Reyes-García A, Colchero MA, Basto-Abreu A (2023). Comparison of Dietary Intake Before vs After Taxes on Sugar-Sweetened Beverages and Nonessential Energy-Dense Foods in Mexico, 2012 to 2018. JAMA Netw Open.

[26] Barrientos-Gutierrez T., Zepeda-Tello R., Rodrigues E.R., Colchero M.A., Rojas-Martínez R., Lazcano-Ponce E. (2017). Expected population weight and diabetes impact of the 1-peso-per-litre tax to sugar sweetened beverages in Mexico. PLOS ONE.

[27] Hernández F.M., Cantoral A., Colchero M.A. (2021). Taxes to unhealthy food and beverages and oral health in Mexico: an observational study. Caries Res.

[28] Guerrero-López C.M., Molina M., Colchero M.A. (2017). Employment changes associated with the introduction of taxes on sugar-sweetened beverages and nonessential energy-dense food in Mexico. Prev. Med..

[29] Roche M., Alvarado M., Sandoval R.C., Gomes F.D.S., Paraje G. (2011). Comparing taxes as a percentage of sugar-sweetened beverage prices in Latin America and the Caribbean. Lancet Reg. Health Am..

[30] Andreyeva T., Marple K., Marinello S., Moore T.E., Powell L.M. (2022). Outcomes following taxation of sugar-sweetened beverages: a systematic review and meta-analysis. JAMA Netw. Open..

[31] White M., Barquera S. (2020). Mexico adopts food warning labels, why now?. Health Syst. Reform..

[32] Taillie LS, Bercholz M, Popkin B, Reyes M, Colchero MA, Corvalán C (2021 Aug). Changes in food purchases after the Chilean policies on food labelling, marketing, and sales in schools: a before and after study. Lancet Planet. Health.

[33] Taillie L.S., Reyes M., Colchero M.A., Popkin B., Corvalán C. (2020). An evaluation of Chile’s law of food labeling and advertising on sugar-sweetened beverage purchases from 2015 to 2017: a before-and-after study. PLOS Med.

[34] Basto-Abreu A., Torres-Alvarez R., Reyes-Sánchez F., González-Morales R., Canto-Osorio F., Colchero M.A. (2020). Predicting obesity reduction after implementing warning labels in Mexico: a modeling study. PLOS Med.

[35] Boyland E., McGale L., Maden M., Hounsome J., Boland A., Angus K. (2022). Association of food and nonalcoholic beverage marketing with children and adolescents’ eating behaviors and health: a systematic review and meta-analysis. JAMA Pediatr.

[36] Rincón-Gallardo Patiño S., Tolentino-Mayo L., Flores Monterrubio E.A., Harris J.L., Vandevijvere S., Rivera J.A. (2016). Nutritional quality of foods and non-alcoholic beverages advertised on Mexican television according to three nutrient profile models. BMC Public Health.

[37] Barquera S., Hernández-Barrera L., Rothenberg S.J., Cifuentes E. (2018). The obesogenic environment around elementary schools: food and beverage marketing to children in two Mexican cities. BMC Public Health.

[38] Márquez I., Tolentino-Mayo L., Barquera S. (2020). Regulación de la publicidad de alimentos y bebidas dirigida a la población infantil: el derecho a la información. Salud Publica Mex.

[39] Nieto C., Espinosa F., Valero-Morales I., Boyland E., Potvin Kent M., Tatlow-Golden M. (2023). Digital food and beverage marketing appealing to children and adolescents: an emerging challenge in Mexico. Pediatr. Obes..

[40] (2020). Modificación a la Norma Oficial Mexicana NOM-051-SCFI/SSA1-2010, Especificaciones generales de etiquetado para alimentos y bebidas no alcohólicas preenvasados.

[41] Hernández-Cordero S., Lozada-Tequeanes A.L., Shamah-Levy T., Lutter C., González de Cosío T., Saturno-Hernández P. (2019). Violations of the International Code of marketing of Breast-milk substitutes in Mexico, Matern. Child Nutr.

[42] Lozada-Tequeanes A.L., Hernández-Cordero S., Shamah-Levy T. (2020). Marketing of breast milk substitutes on the internet and television in Mexico. J. Paediatr. Child Health..

[43] (2014). Acuerdo mediante el cual se establecen los lineamientos generales para el expendio y distribución de los alimentos y bebidas preparados y procesados en las escuelas del Sistema Educativo Nacional.

[44] López-Olmedo N., Jiménez-Aguilar A., Morales-Ruan M.D.C., Hernández-Ávila M., Shamah-Levy T., Rivera-Dommarco J.A. (2018). Consumption of foods and beverages in elementary schools: results of the implementation of the general guidelines for foods and beverages sales in elementary schools in Mexico, stages II and III. Eval. Program Plann..

[45] Pérez-Ferrer C., Barrientos-Gutierrez T., Rivera-Dommarco J.A., Prado-Galbarro F.J., Jiménez-Aguilar A., Morales-Ruán C. (2018). Compliance with nutrition standards in Mexican schools and their effectiveness: a repeated cross-sectional study. BMC Public Health.

[46] Théodore F.L., Moreno-Saracho J.E., Bonvecchio A., Morales-Ruán M.D.C., Tolentino-Mayo L., López-Olmedo N. (2018). Lessons learned and insights from the implementation of a food and physical activity policy to prevent obesity in Mexican schools: an analysis of nationally representative survey results. PLOS ONE.

[47] El Poder del Consumidor (2023). https://elpoderdelconsumidor.org/2023/03/celebramos-votacion-a-favor-de-la-minuta-de-reforma-a-la-ley-general-de-educacion-para-promover-escuelas-saludables-en-la-comision-de-educacion-de-camara-de-senadores/.

[48] Willett W., Rockström J., Loken B., Springmann M., Lang T., Vermeulen S. (2019). Food in the anthropocene: the EAT-Lancet commission on healthy diets from sustainable food systems. Lancet.

[49] Castellanos-Gutiérrez A., Sánchez-Pimienta T.G., Batis C., Willett W., Rivera J.A. (2021). Toward a healthy and sustainable diet in Mexico: where are we and how can we move forward?. Am. J. Clin. Nutr..

[50] Bonvecchio A., Ayala-Niochet M.C., Fernandez Gaxiola A.C., Unar-Munguía M., Théodore F., Rodriguez Ramirez S. (2023).

[51] Mota-Castillo P.J., Unar-Munguía M., Santos-Guzmán A., Ceballos-Rasgado M., Tolentino-Mayo L., Barquera S. (2023). Digital marketing of commercial breastmilk substitutes and baby foods: strategies, and recommendations for its regulation in Mexico, Global. Health.

[52] Batis C., Marrón-Ponce J.A., Stern D., Vandevijvere S., Barquera S., Rivera J.A. (2021). Adoption of healthy and sustainable diets in Mexico does not imply higher expenditure on food. Nat. Food..

[53] Huang T.T.K., Cawley J.H., Ashe M., Costa S.A., Frerichs L.M., Zwicker L. (2015). Mobilisation of public support for policy actions to prevent obesity. Lancet.

[54] Gunningham N., Grabosky P., Sinclair D. (1998).

[55] Howlett M., Halpern L., Pierre L., Gales P.L. (2014). L'instrumentation et ses effets.

[56] Crosbie E., Otero Alvarez M.G., Cao M., Vejar Renteria L.S., Rodriguez E., Larrañaga Flota A. (2023). Implementing front-of-pack nutrition warning labels in Mexico: important lessons for low- and middle-income countries. Public Health Nutr.

[57] Cervantes G., Thow A.M., Gómez-Oliver L., Durán Arenas L., Pérez-Ferrer C. (2021). What opportunities exist for making the food supply nutrition friendly? A policy space analysis in Mexico. Int. J. Health Policy Manag..

[58] Fanzo J., Haddad L., Schneider K.R., Béné C., Covic N.M., Guarin A. (2021). Viewpoint: rigorous monitoring is necessary to guide food system transformation in the countdown to the 2030 global goals. Food Policy.

[59] Soares P., Davó-Blanes M.C., Martinelli S.S., Melgarejo L., Cavalli S.B. (2017). The effect of new purchase criteria on food procurement for the Brazilian school feeding program. Appetite.

[60] Barquera S., Rivera J.A. (2020). Obesity in Mexico: rapid epidemiological transition and food industry interference in health policies. Lancet Diabetes Endocrinol.

[61] Branca F., Demaio A., Udomkesmalee E., Baker P., Aguayo V.M., Barquera S. (2020). A new nutrition manifesto for a new nutrition reality. Lancet.

[62] Nieto C., Valero I., Buenrostro N., Álvarez K., García A., Mendoza B. (2021). Children and adolescents’ exposure to digital food and beverage marketing in Mexico during COVID-19 times. Curr. Dev. Nutr..

